# Single-cell RNA sequencing of immune cells in gastric cancer patients

**DOI:** 10.18632/aging.102774

**Published:** 2020-02-10

**Authors:** Kai Fu, Bingqing Hui, Qian Wang, Chen Lu, Weihong Shi, Zhigang Zhang, Dawei Rong, Betty Zhang, Zhaofeng Tian, Weiwei Tang, Hongyong Cao, Xuehao Wang, Ziyi Chen

**Affiliations:** 1 Department of General Surgery, Nanjing First Hospital, Nanjing Medical University, Nanjing, China; 2 Department of Oncology, The First Affiliated Hospital of Nanjing Medical University, Nanjing, China; 3 Jiangsu Research Center for Primary Health Development and General Education, Jiangsu Vocational College of Medicine, Yancheng, China; 4 Department of General Surgery, Zhongda Hospital, Medical School, Southeast University, Nanjing, China; 5 Michael G. DeGroote School of Medicine, McMaster University, Hamilton, Canada; 6 Department of Laboratory Diagnostics, Changhai Hospital, Second Military Medical University, Shanghai, China; 7 Hepatobiliary/Liver Transplantation Center, The First Affiliated Hospital of Nanjing Medical University, Key Laboratory of Living Donor Transplantation, Chinese Academy of Medical Sciences, Nanjing, China

**Keywords:** gastric cancer, single-cell RNA sequencing, immunotherapy, exhausted

## Abstract

Cancer immunotherapy has achieved positive clinical responses in the treatment of various cancers, including gastric cancer (GC). In this study, we characterized the heterogeneity of T cells isolated from GC patients at the single-cell level using single-cell RNA sequencing. We identified different immune cell subtypes and their heterogeneous transcription factors and depicted their developmental trajectories. In particular, we focused on exhausted CD8^+^ cells and Tregs and discovered that, as compared to control, the IRF8 transcription factor was downregulated in CD8^+^ tumour-infiltrating lymphocytes (TILs) from GC tissues, and that GC patients with lower IRF8 levels in blood CD8^+^ T cells tended to be a at a more advanced disease stage. These findings provide a theoretical basis for targeted immune therapy in GC.

## INTRODUCTION

The wide range of heterogeneity in cancerous tumors has been amply studied to understand its causes and design targeted therapies [1]. While antibodies that block immune checkpoint proteins, including cytotoxic T-lymphocyte associated protein 4 (CTLA4) and programmed cell death protein 1 (PD-1), have been approved to treat a variety of cancers [2], the majority of cancer patients see little benefits from these treatments. One limitation of studies leading to such antibody treatments is their failure to characterize single cells for their ability to respond to checkpoint inhibitors. Indeed, the identification of effective therapeutic bio- markers requires an in-depth understanding of tumor-resident immune cells.

Gastric cancer (GC) is the third leading cause of cancer-related death, with a relatively poor prognosis [3], particularly for patients with tumor, node, metastasis (TNM) stage T3 and T4 [4]. While targeting immune checkpoints has been used with great success to treat some types of cancer and offer great promise to treat GC, GC patients do not benefit much from the current implementation of such therapies.

Recently, single-cell RNA sequencing has enabled specific analysis of cell populations in highly complex tumor micro-environments at the single-cell level, thereby revealing previously uncharacterized molecular complexity [5]. Single-cell analyses might more accurately identify rare gene mutations in tumors as compared to bulk analyses, and might thus facilitate the design of optimal treatments to prevent tumor regeneration [6]. For example, single-cell sequencing has revealed a T cell exhaustion signature in some types of cancer and its connection to T cell activation [7–10]. However, there are no reports of specific applications of single-cell sequencing to GC. In the present study, we analyzed immune cells from a cohort of newly-diagnosed GC patients using flow cytometry and RNA-seq. We also separately analyzed the different genes in different cell clusters from two perspectives: T (gastric cancer tissues) vs N (adjacent normal tissues); PB (gastric cancer peripheral blood) vs HB (healthy individual peripheral blood). We examined signature genes for CD4^+^lymphocytes, CD8^+^ lymphocytes, B lymphocytes, Natural Killer cells (NKs), Dendritic cells (DCs), and macrophages. Our findings provide a theoretical basis for targeted therapy of immune cells in GC and can be used as a valuable resource for studying the basic characteristics of immune cells and potentially guide effective immune-therapy strategies.

## RESULTS

### Acquisition of scRNA-seq profiles from primary GC samples and immune cell clustering

**Figure 1:**
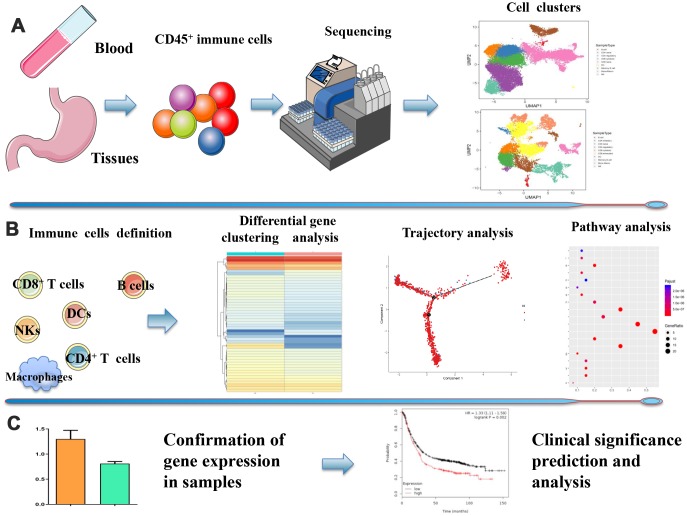
**Overview of the study design.** (**A**) ScRNA-seq was performed on immune cells isolated from GC preoperational peripheral blood samples and GC tissues and corresponding adjacent non-tumor tissues. 10 cell clusters in tissues and 9 cell clusters in peripheral blood were identified based on CD45 isolation. (**B**) Each immune cell subtype, their heterogeneous transcription factors, and their developmental trajectories. (**C**) Correlation between the expression of specific genes and clinical significance.

We performed scRNA-seq on immune cells isolated from nine samples including two peripheral blood samples taken from two healthy individuals, three preoperational peripheral blood samples taken from three GC patients, and two pairs of gastric cancer tissues and corresponding adjacent non-tumor tissues taken from two GC patients. To capture the full spectrum of tumor micro-environments, we sorted a subset of cells with no pre-selection based on CD45 isolation and to ensure adequate numbers of immune cells for analysis. The data separated for by sample are detailed in Table 1. We identified 10 cell clusters in tissues and nine cell clusters in peripheral blood by classifying the cells based on their molecular and functional properties (Figure 1A). Next, we identified each immune cell subtype and their heterogeneous transcription factors (TFs). Figure 1B shows a depiction of their developmental trajectories (Figure 1B). Finally, we confirmed the expression of some genes and analyzed its correlation with clinical features (Figure 1C).

**Table 1:** The sample information of patients.

**Sample ID**	**Age**	**Sex**	**TNM stage**	**Type**	**Cell Number**
RD20180928003	69	Male	IIIA	T1	1681
RD20180928004	69	Male	IIIA	N1	3037
RD20181119022	67	Female	IIB	T2	2505
RD20181119023	67	Female	IIB	N2	2505
RD20181018007	61	Male	IIIA	PB1	377
RD20181018008	71	Male	IIIA	PB2	1430
RD20181109021	83	Male	IIB	PB3	4154
RD20181018009	65	Male	-	HB1	6373
RD20181018010	72	Female	-	HB2	7333

Note: T: Tissue; N: Normal; PB: Peripheral blood of cancer patients; HB: Blood of healthy individuals

### IRF8 is downregulated in exhausted CD8^+^ T cells from GC samples compared to normal samples

**Figure 2:**
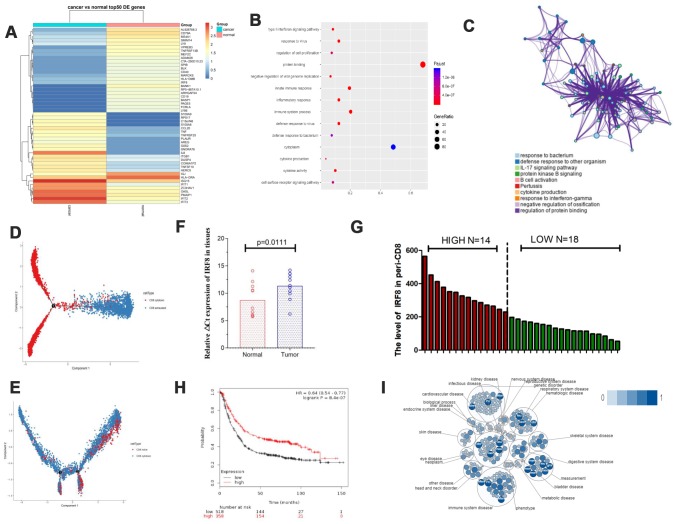
**The transcription factor IRF8 was associated with CD8^+^ T cells in GC.** (**A**) Heat map displaying the top 50 genes differentially expressed in CD8^+^ exhausted T cells from tissues. (**B** and **C**) Pathway analysis for CD8^+^ exhausted T cells. (**D**) Trajectory analysis for CD8^+^ T cells in tissues. (**E**). Trajectory analysis for CD8^+^ T cells in blood. (**F**) Expression of IRF8 in CD8^+^TILs from GC tissues and normal tissues. (**G**) Expression of IRF8 in peripheral blood CD8^+^ T cells from GC patients. (**H**) TGCA analysis of IRF8 in GC prognosis. (**I**). Pathway and disease analysis of IRF8.

To reveal the intrinsic structure and potential functional sub-types of CD8^+^ T cells including naive, cytotoxic, and exhausted CD8^+^ T cells, we separately analyzed the genes in T vs N and PB vs HB groups and made heat maps and volcano formats (Figures 2A, 2B and Supplementary Figures 1, 2). Since co-inhibitory receptors, such as PDCD1 and TIGIT, are targets for cancer immunotherapies, we then focused on analyzing the preferential enrichment of exhausted CD8^+^ T cells in GC. Pathway analysis showed that these different genes in tumor-infiltrate exhausted CD8^+^ T cells might be involved in cytokine production (Figure 2C). In terms of trajectory branch, we observed that it started with cytotoxic CD8^+^ T cells and ended with exhausted CD8^+^ T cells in GC tissues (Figure 2D) while in blood it ended with naive CD8^+^ T cells (Figure 2E), which is consistent with the normal process of tumors.

We assessed the expression of the interferon regulatory factor 8 (IRF8) transcription factor in tumor-infiltrate CD8^+^ exhausted T cells and CD8^+^ TILs from 11 GC patient tumors and found that IRF8 was downregulated in both compared to normal tissues (Figure 2F). Furthermore, we analyzed peripheral blood mononuclear cells (PBMCs) from 32 patients with GC at initial diagnosis to assess IRF8 expression in CD8^+^ T cells using flow cytometry (Figure 2G). We divided GC patients into Peri-CD8-IRF8 high and Peri-CD8-IRF8 low groups according to the average levels of IRF8 in peripheral blood CD8^+^ T cells. GC patients with low expression of IRF8 in blood CD8^+^ T cells had a more advanced tumor stage (Table 2). Based on GC data from The Cancer Genome Atlas (TCGA), downregulation of IRF8 was associated with shorter overall survival (OS) (Figure 2H). Open Targets software showed that IRF8 plays a negative regulatory role in cells of the immune system (Figure 2I). Together, these data indicate that IRF8 was associated with exhausted CD8^+^ T cells in GC.

**Table 2:** Clinical and pathological features of two groups of patients with Peri-CD8-IRF8^high^ and Peri-CD8-IRF8 ^lo^^w^

**Variables**	**No. of patients**	**Peri-CD8-IRF8 ^high^**	**Peri-CD8-IRF8 ^low^**	**P value**
**Age(year)**				0.252
≥60	15	8	7	
<60	17	6	11	
**Gender**				0.292
Female	12	4	8	
Male	20	10	10	
**Diameter**				0.590
≥5(cm)	21	9	12	
<5(cm)	11	5	6	
**Differentiation**				0.361
High	16	6	10	
Low/Middle	16	8	8	
**TNM Stage**				0.017*
I–II	15	10	5	
III	17	4	13	

Note: *P<0.05.

### Identification of genes uniquely associated with Treg function in GC

Regulatory T cells (Tregs) are involved in immune tolerance [11]. Supplementary Figure 3A, 3B shows different gene and pathway analyses in blood-isolated Tregs. Great effort has been devoted to identifying genes that can serve as to monitor GC prognosis. Our results showed that KDM5D and ADRB2 in blood-isolated Tregs was upregulated in peripheral blood of GC patients compared to controls and that high expression of KDM5D and ADRB2 correlated with poor prognosis (Supplementary Figure 3C, 3D). This suggests that KDM5D and ADRB2 might serve as non-invasive markers in circulating blood to monitor GC patient outcomes.

**Figure 3:**
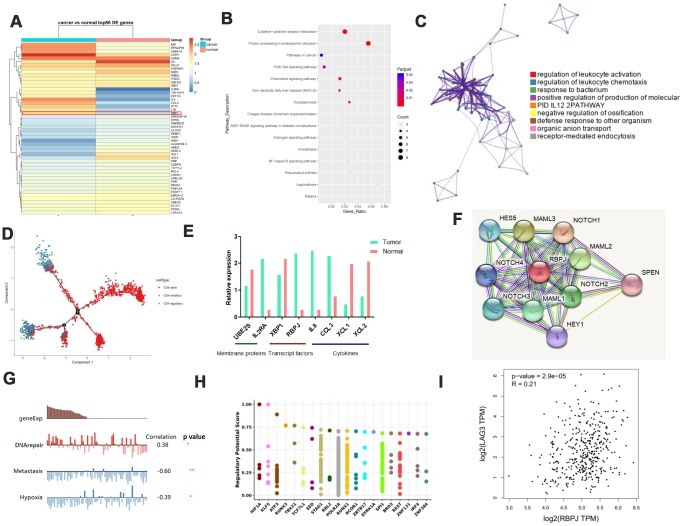
**Identification of genes uniquely associated with Treg function in GC.** (**A**) Heat map displaying the top 50 genes differentially expressed in Tregs from tissues. (**B** and **C**) Pathway analysis for different genes in Tregs. (**D**) Trajectory analysis for Tregs in tissues. (**E**) Expression of various molecules in Tregs. (**F**) STRING analysis of RBPJ. (**G**) Single-cell analysis using CancerSEA. (**H**) Top 20 differentially expressed TFs in cancers as shown by Cistrome DB Toolkit for RBPJ. (**I**) GEPIA analyses showing the association between RBPJ and LAG3.

In tumor-infiltrate Tregs, we found that IFIT2, CCL3, RBPJ, etc. were upregulated in GC tissues compared to adjacent normal tissues while IGJ, XCL1, XCL2, etc. were downregulated (Figure 3A). Pathway indicated they were enriched in cytokine-cytokine receptor interaction, PI3K-AKT, and NF-kB pathway (Figure 3B, 3C). For trajectory branch, we observed much fewer effector Tregs in the tumor environment but more pronounced naive phenotypes in GC tissues (Figure 3D). We highlight some membrane receptors, transcript factors, and cytokines that are differentially expressed in GC. Interestingly, we found that the transcription factor RBPJ was overexpressed in tumor-infiltrate Tregs (Figure 3E). Interaction network analysis using STRING displayed that RBPJ was involved in the NOTCH pathway (Figure 3F). Next, we performed single-cell analysis using CancerSEA. The results suggested that RBPJ might function mainly via regulating DNA repair, metastasis, and hypoxia to inhibit cancer progression (Figure 3G). To explore the potential network which could regulate RBPJ expression, we analyzed the TFs that might promote or inhibit RBPJ gene transcription. We found the top 20 TFs differentially expressed in cancer tissues using the Cistrome DB Toolkit (Figure 3H). We found that RBPJ might act as a TF to promote LAG3 and GEPIA2 expression in GC (Figure 3I, Supplementary Figure 4E).

### Gene signature of B cells and pathway analysis in GC

**Figure 4:**
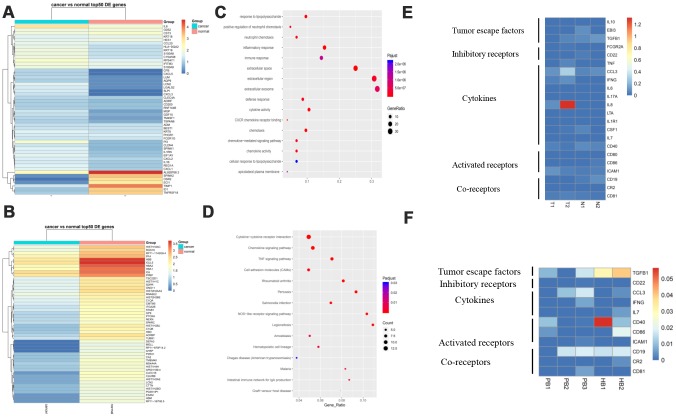
**Gene signature of B cells and pathway analysis.** (**A**) The expression analysis of functional molecules in B cell cluster in T vs N. (**B**) The expression analysis of functional molecules in B cell cluster in PB vs HB. (**C**) Pathway analysis of in B cell cluster in T vs N. (**D**) Pathway analysis of in B cell cluster in PB vs HB. (**E**) The expression analysis of functional molecules in B cell cluster in T vs N. (**F**) The expression analysis of functional molecules in B cell cluster in PB vs HB.

Differentially expressed genes in B cell subsets between cancerous and paracancerous tissues was comprised of EIF1AY, KRT19, LCN2, RPS4Y1, etc., (Figure 4A). In the PB vs HB group, RORA, COL6A2, ETS1, FHIT, etc., were overexpressed while HBA2, HBA1, IGLL5, C1CB, etc., were downregulated (Figure 4B). Pathway analysis revealed that upregulated genes in the B cell cluster were scattered across the TNF, NOD-like, and CXCR chemokine receptor binding pathways (Figure 4C, 4D). It is worth mentioning that we summarized our current knowledge of B cells and performed heat-maps in T vs N and PB vs HB group. The results revealed that B cells exert immune-regulatory functions through the production of cytokines including IFNG, CCL3, IL-8, etc. We also presented a succinct summary of emerging immune targets with reported pre-clinical efficacy including activated/ inhibitory/co-receptors of B cells [12]. For example, CD40, which serves as a B cell activating receptor, was downregulated in GC patients (Figure 4E, 4F).

### More inhibitory receptors and less activated receptors secreted by NK cells in response to GC

**Figure 5:**
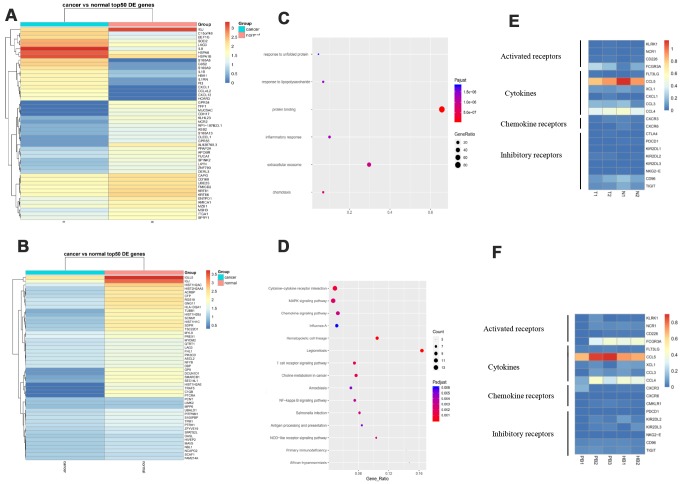
**More inhibitory receptors and fewer activated receptors are secreted by NK cells in response to GC.** (**A**). Expression analysis of functional molecules in the NK cell cluster in T vs N. (**B**). Expression analysis of functional molecules in the NK cell cluster in PB vs HB. (**C**). Pathway analysis of functional molecules in the NK cell cluster in T vs N. (**D**). Pathway analysis of functional molecules in the NK cell cluster in PB vs HB. (**E**). Expression analysis of functional molecules in the NK cell cluster in T vs N. (**F**). Expression analysis of functional molecules in the NK cell cluster in PB vs HB.

NK cells are important components of the innate immunity and play a key role in host defense by virtue of their ability to release cytokines and to mediate cytolytic activity against tumor cells [13]. In our research, we found that IL8, G0S2, HSPA6, CXCL1, etc., were upregulated in GC tissues compared to adjacent normal tissues while IGJ, TFF1, NCR2, etc. were downregulated (Figure 5A). In the PB vs HB group, SCAF1, LAG3, TPM1, etc. were overexpressed whereas IGLL5, IGJ, C1QB, etc. were overexpressed (Figure 5B). Pathway analysis demonstrated that these genes might participate in cytokine-cytokine receptor interaction, MAPK signaling, chemokine signaling, and T cell receptor signaling (Figure 5C, 5D). Of note, NK cells expressed more inhibitory receptors such as KIR2DL2 in GC tissues compared to controls. Similarly, NK cells expressed fewer activated receptors such as KLRK1 and CD226 in GC patient blood compared to controls. Lastly, NK cells secreted more cytokines including CCL3 and CCL4 in GC (Figure 5E, 5F). Taken together, these data indicate that NK cells participate actively in immunosurveillance to prevent GC.

### Different dendritic cell subtypes and their interactions in GC

**Figure 6:**
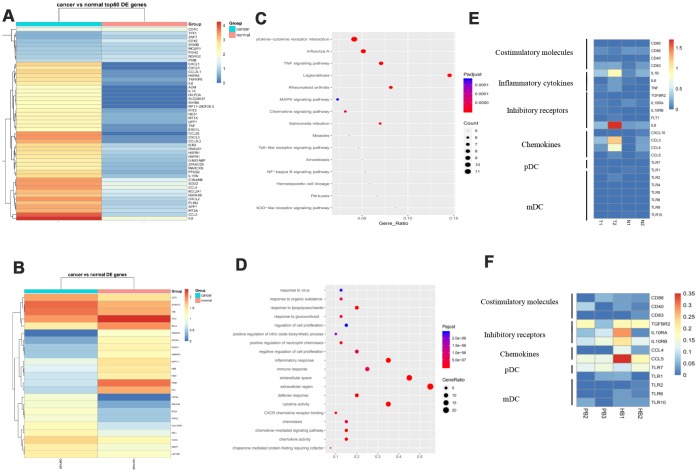
**Different DC subtypes and their interactions in GC.** (**A**) Expression analysis of functional molecules in the DC cell cluster in T vs N. (**B**) Expression analysis of functional molecules in the DC cell cluster in PB vs HB. (**C**) Pathway analysis of functional molecules in the DC cell cluster in T vs N. (**D**) Pathway analysis of functional molecules in the DCB cell cluster in PB vs HB. (**E**) Expression analysis of functional molecules in the DC cell cluster in T vs N. (**F**) Expression analysis of functional molecules in the DC cell cluster in PB vs HB.

Dendritic cells (DCs) are central regulators of the adaptive immune response, and as such are necessary for T-cell-mediated cancer immunity [14]. Figure 6A, 6B lists differentially expressed genes in the DC cell cluster (Figure 6A, 6B). Pathway analysis suggested that these genes are enriched in cytokine activity and immune response, MAPK, and NF-KB pathways (Figure 6C, 6D). Plasmacytoid DCs (pDCs) are recognized as major producers of type I interferons (IFN-I) and can promote anti-tumoral immunity through direct activity on both tumors and immune cells [15]. pDCs selectively express TLR7 and TLR9, and their most important function is to produce large quantities of IFN-I in response to single-stranded viral RNA and DNA [16]. Our data showed that pDCs expressed more TLR7 in GC patients than in controls. Additionally, DC cells expressed more inhibitory receptors such as FTL and IL8 in GC tissues compared to normal tissues and secreted less cytokines including CCL4 and CCL5 in GC patient blood compared to controls. Myeloid DCs (mDCs) are very potent antigen-presenting cells (APCs) that possess the unique capacity to prime naive T cells and consequently to initiate a primary adaptive immune response [17]. We showed that some molecules including TLR1, TLR2, TLR6, and TLR10 are differentially expressed in mDCs (Figure 6E, 6F). The complexity of DC subtypes, and their interactions, means that multiple complementary strategies are likely necessary to drive the eradication of cancer in GC patients undergoing DC-mediated anti-cancer therapy.

### Gene signature of macrophages in GC

Macrophages are a major constituent of the tumor microenvironment where they either promote or inhibit tumorigenesis and metastasis depending on their state [18]. Pathway analysis failed to identify differentially-expressed genes in macrophage subsets (Supplementary Figure 4A, 4B). We listed some key inflammatory cytokines, inhibitory receptors, angiogenesis factors, chemokines, inhibition of angiogenic factors, growth factors, and others in three groups and made heat-maps. Results showed that macrophages expressed more IL1RN, CXCL1, and IL8 in GC tissues and more CCL5, IL2RG, and C10orf54 in GC blood than in controls (Supplementary Figure 4A, 4B). Plasticity is a hallmark of cells of the myelomonocytic lineage [19]. A better understanding of the molecular basis of macrophage plasticity will open new vistas in immunopathology and therapeutic interventions for GC.

## DISCUSSION

Intratumoral heterogeneity is a major challenge in oncology but scRNA-seq is an emerging efficient tool to characterize tumor developmental levels, drug resistance, and invasiveness. Here, we provide a comprehensive overview of immune cells in human gastric cancer (GC) tissues and GC preoperative blood at a single-cell resolution. Our findings support the view that immune cells from different tissues and blood are instructed by environmental factors to display different gene-expression profiles. Previous studies mostly focused on the expression profile of T cell populations in various cancers including liver cancer [20], breast cancer [21], and melanoma [22]. However, our data identified the distribution of immune cell clusters including CD4^+^, CD8^+^ T cells, B cells, NK cells, DC cells, and macrophages in GC, their characteristic gene expression, and pathway analyses in T vs N and PB vs HB groups.

Immunization is caused by a complex interaction between the innate immune system and the adaptive immune system. Innate immunity is the first line of defense against infection and abnormal cells. Importantly, NK cells, DC cells, and macrophages serve as important antigen-presenting cells that activate adaptive immunity [13, 23, 24]. Therefore, we analyzed innate immunity and listed key inflammatory cytokines, inhibitory/activated receptors, angiogenesis factors, chemokines, inhibition of angiogenic factors, growth factors, and others in NK cells, DC cells, and macrophages, which were presented as heat-maps. The success of anti-cancer immunotherapy has placed NK cells, DC cells, and macrophages under the spotlight, given their critical role in initiating anti-tumor T cell immunity. Looking forward, the development of novel immunotherapeutic interventions for gastric cancer should aim to enhance the function of tumor-associated NK cells, DC cells, and macrophages to improve GC patient outcomes and exploit this critical immune cell type.

Transcription factors are key regulators of gene expression from DNA to mRNA by binding to specific DNA sequences on promoters and enhancers to activate or inhibit the expression of specific genes [25]. Therefore, the identification of target genes for TFs is very important in understanding normal development and disease pathogenesis. A central finding from our study is that IRF8 was downregulated in tumor-infiltrate CD8^+^ exhausted T cells compared to adjacent normal tissues. IRF8 was also downregulated in CD8^+^ TILs from GC tumors compared to normal tissues. GC patients with low expression of IRF8 in blood CD8^+^ T cells had a more advanced tumor stage. These data indicate that IRF8 was associated with exhausted CD8^+^ T cells in GC. Miyagawa F et al showed that IRF8 integrates the TCR/costimulation and γc-cytokine-signaling pathways and mediates the transition of naive CD8 T cells to effector cells, thus identifying IRF8 as a regulator of CD8 T-cell differentiation [26]. Most previous studies have focused on IRF8’s role as a transcription factor in DCs. For example, Luda et al found that IRF8 Transcription-Factor-Dependent Classical Dendritic Cells are essential for intestinal T cell homeostasis [27]. Sichien et al 2016 identified IRF8 as a terminal selector of the cDC1 lineage controlling survival. In monocytes, IRF8 was necessary during early but not late development. Complete or late deletion of IRF8 had no effect on pDC development or survival but altered their phenotype and gene- expression profile leading to increased T cell stimulatory function but decreased type 1 interferon production [28]. Our results here show a novel mechanism for IRF8-mediated tumor CD8^+^ T cell activation. In addition, we found that another transcription factor, RBPJ, was overexpressed in tumor-infiltrate Tregs and might regulate the LAG3. While it is known that RBPJ deficiencies can lead to splenomegaly, lymphadenopathy, the spontaneous formation of germinal centers, and a TH2-associated immunoglobulin class switch [29], RBPJ’s contributions to cancer remain poorly understood.

The adaptive immune system of the human body mainly relies on the T cell receptor (TCR) and specific binding of the complementarity-determining region on the B cell receptor (BCR) to antigen peptides. ScRNA-seq is a powerful tool for defining TCR sequences per cell and can be used to identify adaptive complexes (MHCs) of viral antigens or tumor-specific new antigenic tumor cells [30]. The limitation of our study is that we have not used immunohistochemical high-throughput immunoassay library sequencing to deeply sequence the complementarity-determining regions of B cell receptors and T cell receptors. Therefore, future studies should combine expression and TCR/BCR-based analyses to reveal the connectivity and potential developmental paths of these subsets. Nonetheless, our comprehensive single cell database here is a detailed characterization of GC immune cells from tissues and blood, in term of their clustering, dynamics, and developmental trajectory, as well as unique expression profiles, highlighting potential therapeutic targets for GC such as the transcription factor IRF8.

## MATERIALS AND METHODS

### Patients

Samples were obtained from Department of General Surgery in Nanjing First Hospital and Zhongda Hospital. The cancer tissue samples were derived from two GC patients with untreated, primary, non-metastatic gastric tumors that underwent GC resection. The adjacent normal gastric tissues were taken more than 5 cm away from the cancerous tissues. Peripheral blood samples were obtained from three GC patients before surgery. Two healthy samples of 10 ml normal venous blood were obtained from the individuals without any underlying diseases in physical examination center of Nanjing First Hospital in accordance with the Helsinki Declaration.

### Tissue and blood processing

Gastric tissues were taken by pathologists from normal and tumor regions. Tissues were sliced into small pieces and put into a gentle MACS C Tube (Miltenyi Biotec; 130-093-237) containing 200 μL Enzyme H, 100uL Enzyme R, 25uL Enzyme A (all provided in the human tumor dissociation kit [Miltenyi Biotec; 130-095-929]), and 4.7ml RPMI 1640 (Gibco; 8117133). The C tube was processed on a gentle MACS Octo Dissociator with Heaters (Miltenyi Biotec; 130-096-427) using the program “37C_h_TDK_2” for 1h. The resulting suspension was passed through a 70 μm cells strainer (Miltenyi Biotec; 130-098-462) and washed with 1X PBS containing 0.04% BSA. Live cells were enriched using a Dead Cell Removal kit (Miltenyi Biotec; 130-090-101) as per the manufacturer’s instructions. Enriched live cells were washed with and counted using a hemocytometer with trypan blue. Peripheral blood mononuclear cells (PBMCs) were isolated from blood using a Ficoll-Paque Plus (GE;17-1440-02) according to the manufacturer’s instructions.

### Flow cytometry and cell sorting of samples

The antibodies used for cell surface labeling were Hu CD45 PE HI30 (BD; 555483). Cells were labeled for 45 min at 4 °C while protected from light. Sorting of single cells was performed on BD FACS Aria III instrument, with specific forward and side scatter settings to select for immune cells and exclude doublets. The data we analyzed with FlowJo 10.0.7 software.

### 10X genomics scRNA-seq

The concentrations of single cell suspensions were manually counted using a hemocytometer. Cells were loaded according to standard protocol of the Chromium single cell 3’ kit in order to capture between 3000/5000 cells/chip position (V2 chemistry). Single-cell capture, reverse transcription, cell lysis, and library preparation were performed per the manufacturer’s protocol. Sequencing was performed on HiSeq 4000 (Illumina, 150-bp paired-end protocol).

### Data analysis

SCANPY is a scalable toolkit for analyzing single-cell gene expression data. It includes methods for pre-processing, visualization, clustering, pseudotime and trajectory inference, differential expression testing, and simulation of gene regulatory networks. Its Python-based implementation efficiently deals with data sets of more than one million cells (https://github.com/theislab/Scanpy) [31]

### Definition and grouping criteria of CD8^+^ exhausted T cells in GC

CD8^+^ exhausted T cells are highly expressed on the surface of some inhibitory molecules, such as PD-1, CTLA4, LAG3 (lymphocyte-activation gene-3), and TIGIT (T cell immunoreceptor with Ig and ITIM domains). These inhibitory surface molecules, combined with corresponding ligands on the surface of tumor cells, inhibit the killing capacity of CD8^+^ T cells, leading to tumor immune escape.

### Online prediction software

We used KaplanMeier Plotter (http://kmplot.com/analysis/index.php?p=background) to examine correlations between gene expression and prognosis of cancer [32]. CancerSEA (http://biocc.hrbmu.edu.cn/CancerSEA/) was used to explore the potential roles of IRF8 in cancer [33]. Metascape (http://metascape.org/gp/index.html#/main/step1) is a web-based portal that combines functional enrichment, interactome analysis, gene annotation, and membership search [34]. STRING (https://string-db.org/cgi/input.pl) is a database of known and predicted protein-protein interactions. We utilized STRING to create an interaction network between IRF8 and other important proteins [35]. The Cistrome DB Toolkit database (http://dbtoolkit.cistrome.org) allows users to query transcription factors (TFs) that might regulate genes of interest to identify binding factors, histone modifications, and chromatin accessibility in a genomic interval of interest up to 2 Mb in length [36]. GEPIA2 (http://gepia2.cancer-pku.cn/#index) provides tumor/normal differential expression analysis, correlation analysis, and dimensionality reduction analysis.

### Confirmation of IRF8

PBMCs isolated from patients with GCs or HVs (healthy volunteers) were stained with the following antibodies: FITC anti-human CD3(BD Biosciences), APC anti-human CD8 (BD Biosciences), PE anti-human IRF8 (BD Biosciences). The molecular phenotypes of peripheral blood leucocytes were analyzed immediately by flow cytometry (BD FACS Canto™ II) using the FlowJo V10 (Tree Star) software. CD8^+^ TIL cells were isolated from GC tissues using a human CD8^+^ T cell isolation kit (#17953, Stem cell) following the manufacturer’s instructions. QRT-PCR reactions were performed to detect the expression of IRF8 using the ABI7500 System and the SYBR Green PCR Master Mix (TaKaRa). The primers used were IRF8 F: 5′-TCCGGATCCCTTGGAAACAC and IRF8 R: 5′-CCTCAGGAACAATTCGGTAA. GAPDH was used as a control.

### Statistical analysis

Data are presented as mean ± standard error of the mean (SEM). Statistical analyses and graphic presentation were carried out using the GraphPad Prism version 5.0 (GraphPad Software, San Diego, CA, USA). A *t*-test was used if a normality test was passed; otherwise, the nonparametric Mann-Whitney test was used to analyse the data. Similarly, the Pearson method or the nonparametric Spearman method was used for correlation analyses. Regarding cut-off values, P < 0.05 was considered statistically significant.

### Ethics approval and consent to participate

The human cancer tissues used in this study were approved by the Ethics Committee of Nanjing First Hospital and Ethics Committee of Zhongda Hospital.

## Supplementary Material

Supplementary Figures
