# Supplementary material for: Single-cell RNA sequencing of immune cells in gastric cancer patients
Source: Aging (Albany NY). 2020 Feb 10;12(3):2747–63. doi: 10.18632/aging.102774 (PMC7041746; doi:10.18632/aging.102774)
Supplement: Supplementary Figures [file aging-12-102774-s001..pdf]

SUPPLEMENTARY FIGURES

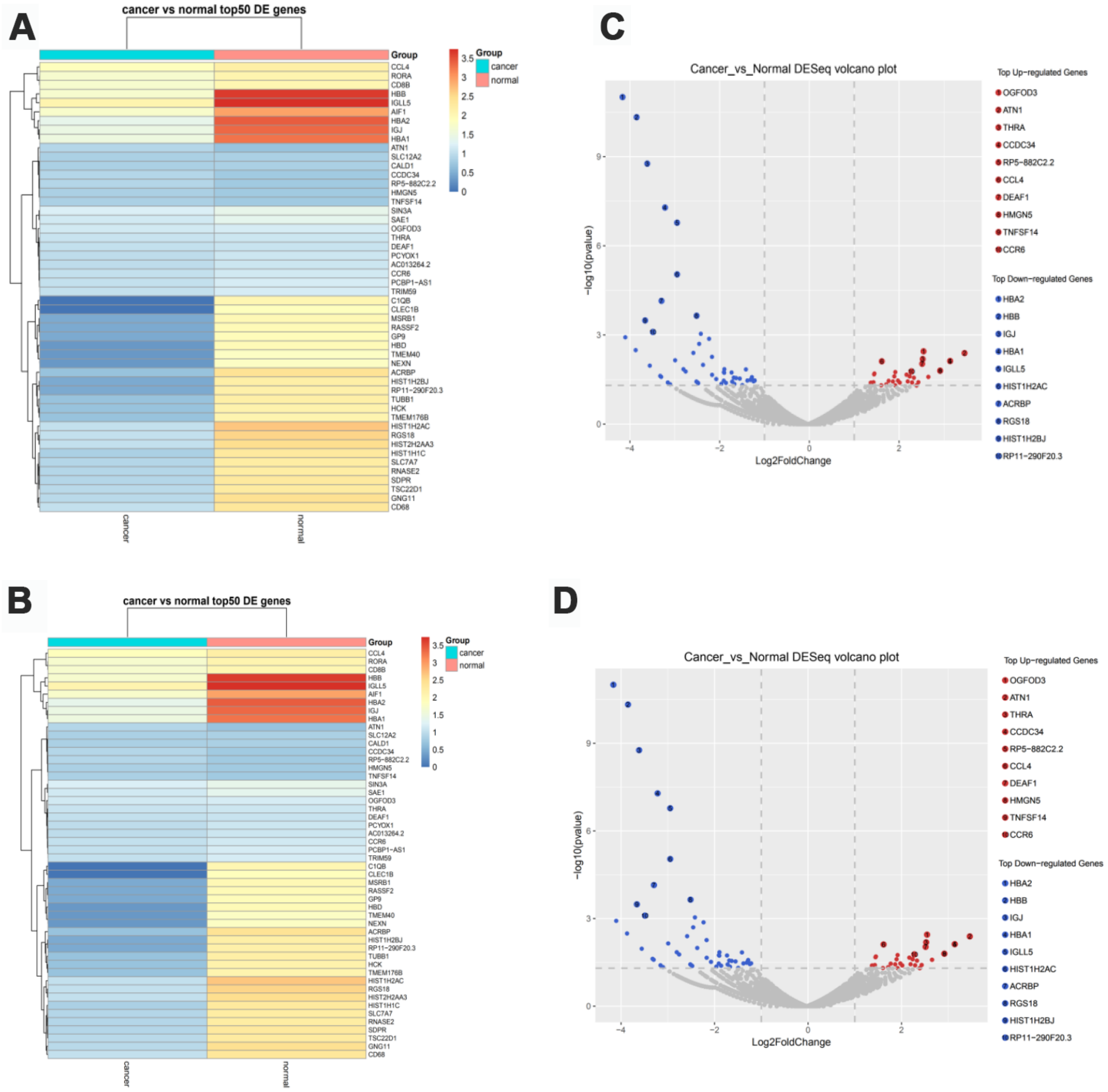

Supplementary Figure 1. Identification of CD8$^+$ naive T cells in GC. (**A**) Heat map displaying the top 50 different genes in CD8$^+$ naive T cells in T vs N. (**B**) Heat map displaying the top 50 different genes in CD8$^+$ naive T cells in PB vs HB. (**C**) Volcano plot showing the top 10 upregulated and 10 downregulated genes in CD8$^+$ naive T cells in T vs N. (**D**) Volcano plot showing the top 10 upregulated and 10 downregulated genes in CD8$^+$ naive T cells in PB vs HB.

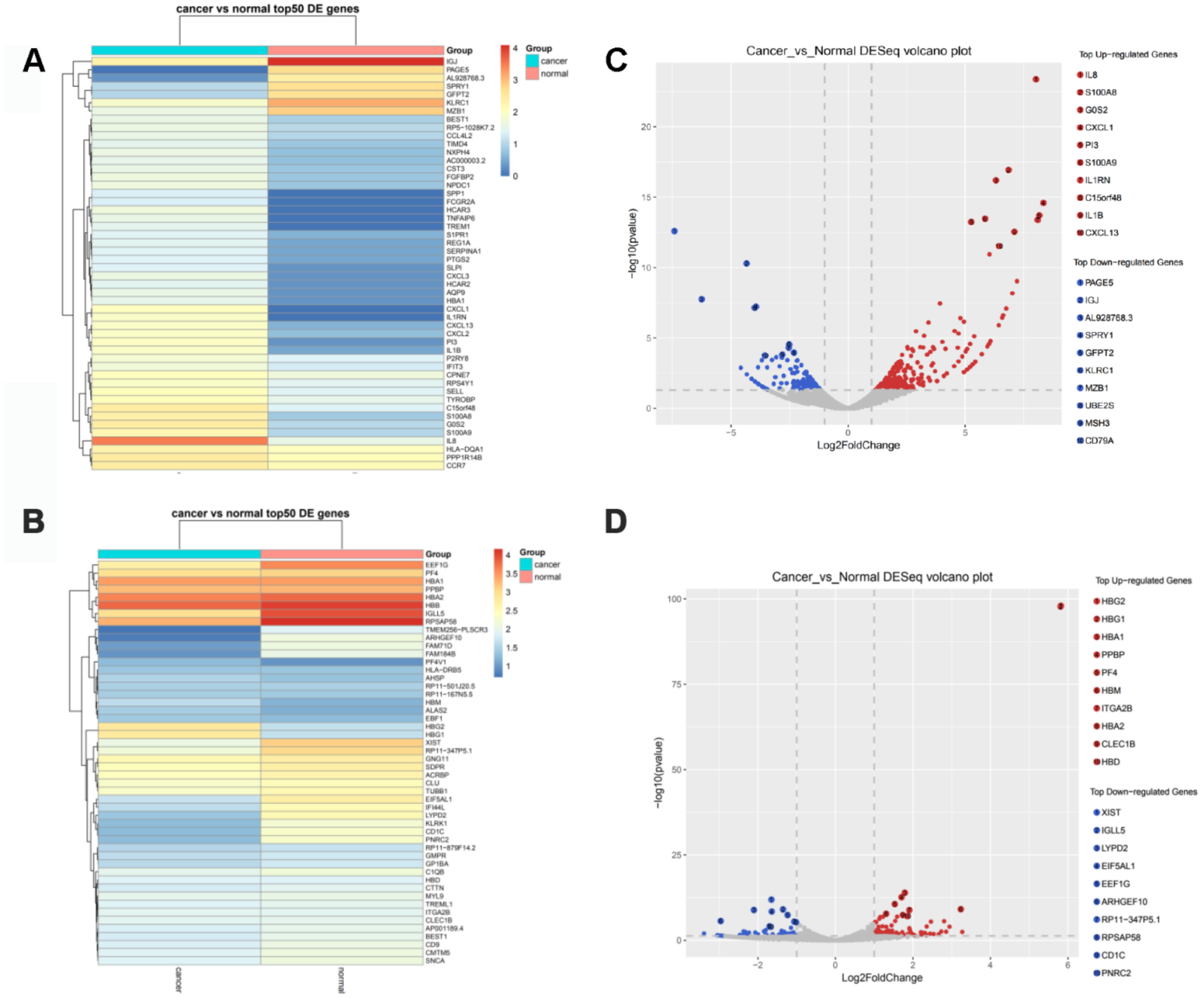

Supplementary Figure 2. Identification of CD8$^+$ cytotoxic T cells in GC. (**A**) Heat map displaying the top 50 differentially expressed genes in CD8$^+$ cytotoxic T cells in T vs N. (**B**) Heat map displaying the top 50 differentially expressed genes in CD8$^+$ cytotoxic T cells in PB vs HB. (**C**) Volcano plot showing the top 10 upregulated and 10 downregulated genes in CD8$^+$ cytotoxic T cells in T vs N. (**D**) Volcano plot showing the top 10 up-regulated and 10 down-regulated genes in CD8$^+$ cytotoxic T cells in PB vs HB.

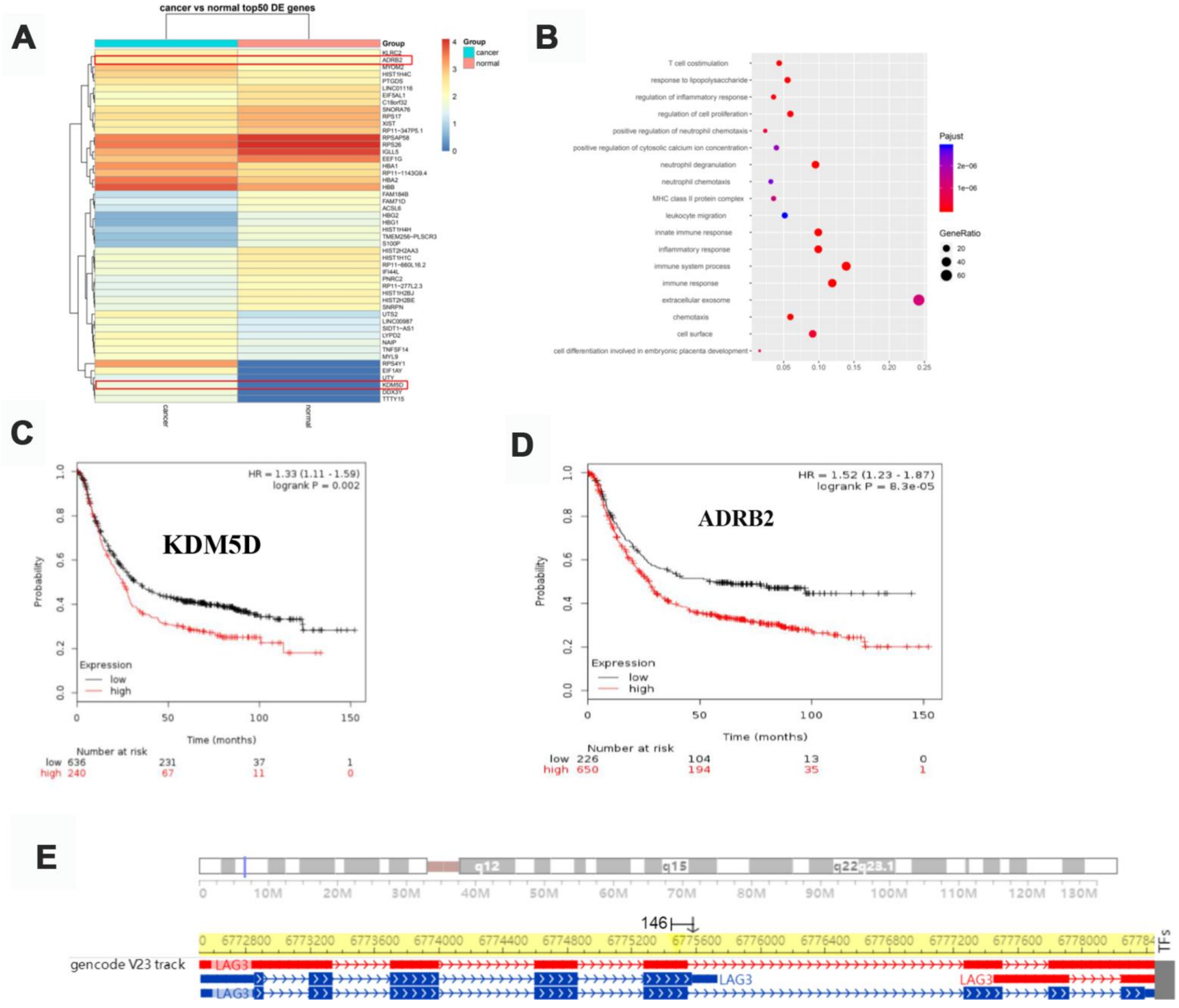

Supplementary Figure 3. Identification of Tregs in GC blood. (**A**) Heat map displaying the top 50 differential genes in Tregs from blood. (**B**) Pathway analysis for different genes in Tregs. (**C** and **D**) KM-plotter database analysis of KDM5D and ADRB2 in GC. (**E**) RBPJ regulates the locus of LAG3.

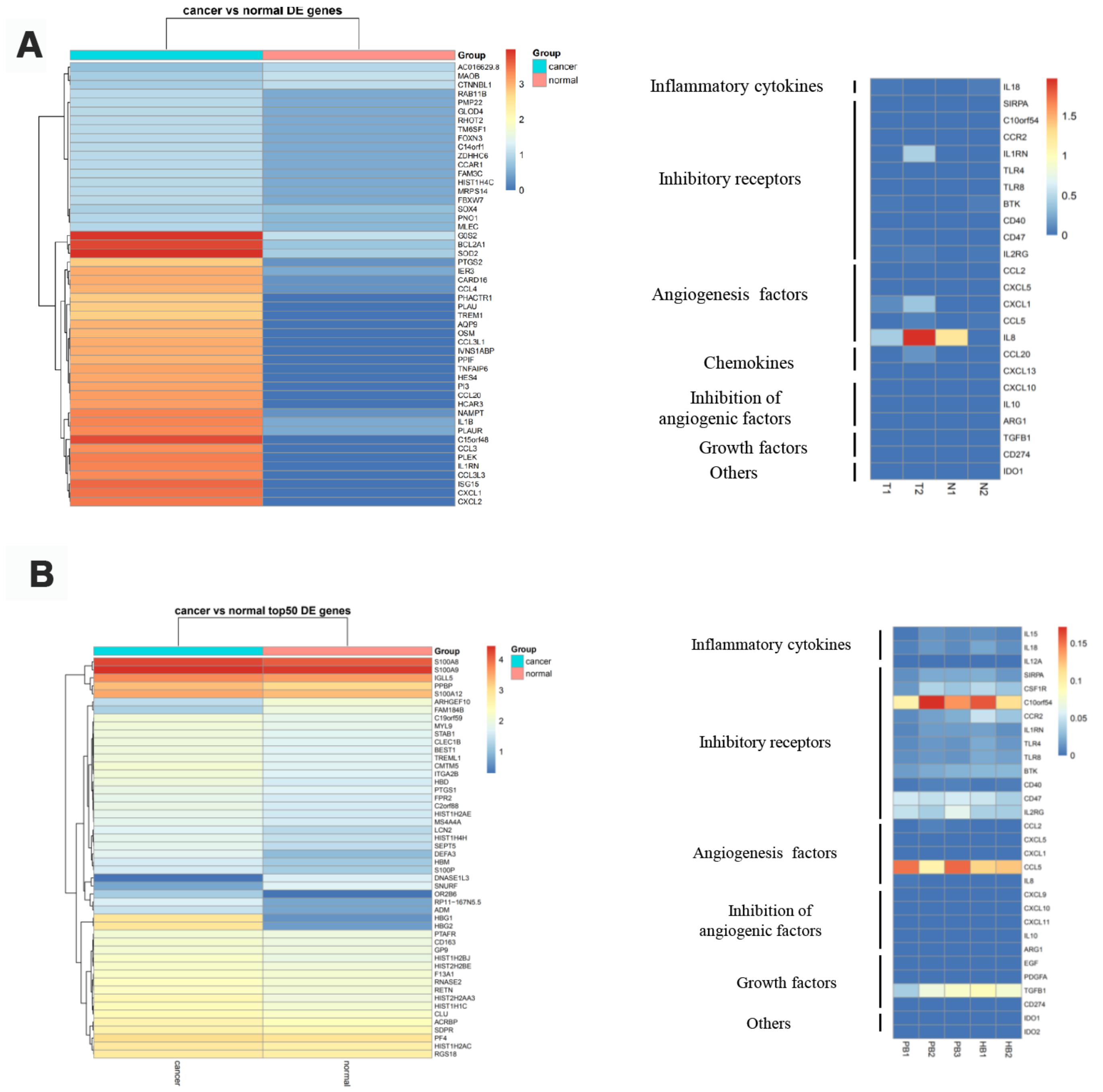

Supplementary Figure 4. Gene signature of macrophages and pathway analysis. (**A**) The expression analysis of functional molecules in macrophages cell cluster in T vs N. (**B**) The expression analysis of functional molecules in macrophages cell cluster in PB vs HB
